# Supplementary figures and images for: ACSA‐2 and GLAST classify subpopulations of multipotent and glial‐restricted cerebellar precursors
Source: J Neurosci Res. 2021 May 31;99(9):2228–49. doi: 10.1002/jnr.24842 (PMC8453861; doi:10.1002/jnr.24842)

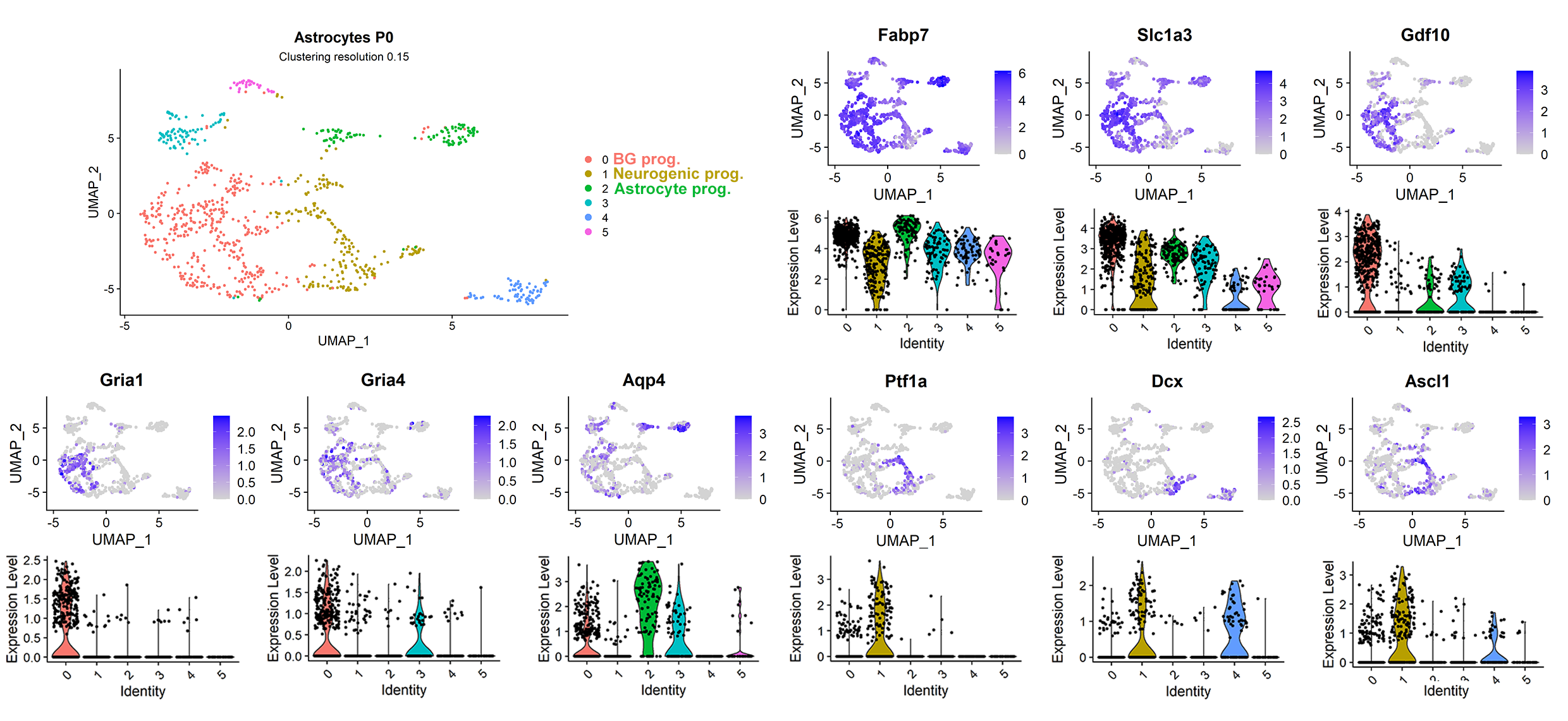

Supplement: Supplementary file 1 — FIGURE S1 Identification of distinct astrocyte subpopulations in scRNA‐seq data sets of P0 cerebella. Seurat clustering of the astrocyte‐like cells in a data set of P0 cerebella resulted in clearly segregated subpopulations that could be classified according to the expression of known marker genes (see Methods). The expression of BG‐enriched genes such as Gdf10, Gria1, and Gria4 allowed to identify the BG/BG progenitor subpopulations (cluster n. 0). The expression of Aqp4 was used to identify the parenchymal astrocytes/astrocyte progenitor population (cluster n. 2). Moreover, the comparison of the gene expression signatures of each of these two populations across ages (see also Figures S2 and S3) confirmed the classification as a good fit. At P0, cluster n.1 expressed genes typically associated with a neuronal progenitor fate, such as Ptf1a, Dcx, and Ascl1 (Hoshino et al., 2005; Gleeson et al., 1999, Grimaldi et al., 2009), and was therefore classified as a cluster of neurogenic progenitors. Further clusters did not present a clear identity—based on known marker genes—and were therefore not further considered for the analyses [file JNR-99-2228-s007.tif]

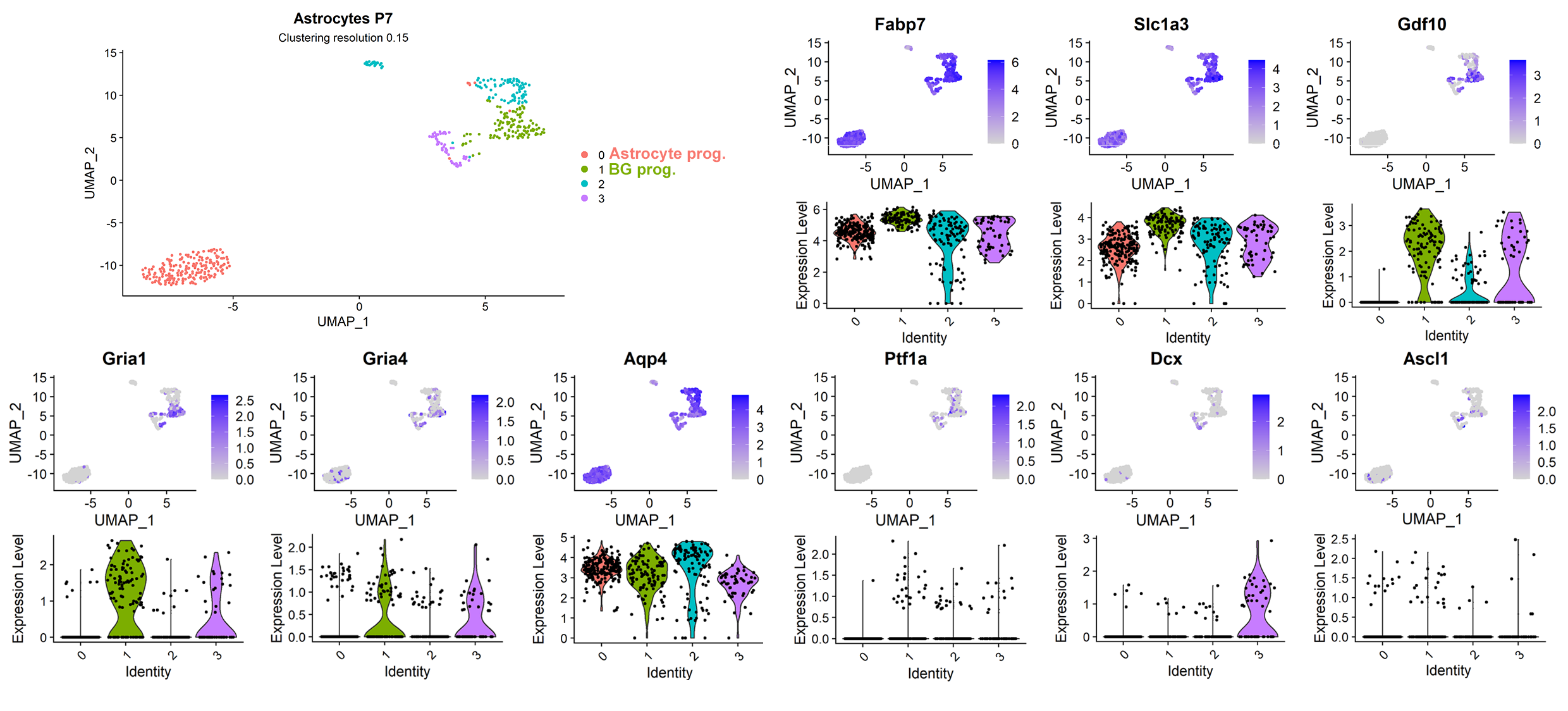

Supplement: Supplementary file 2 — FIGURE S2 Identification of distinct astrocyte subpopulations in scRNA‐seq data sets of P7 cerebella. Seurat clustering of the astrocyte‐like cells in a data set of P7 cerebella resulted in clearly segregated subpopulations that could be classified according to the expression of known marker genes (see Methods). The expression of BG‐enriched genes such as Gdf10, Gria1, and Gria4 allowed to identify the BG/BG progenitor subpopulations (cluster n.1). The expression of Aqp4 was used to identify the parenchymal astrocytes/astrocyte progenitor population (cluster n.0). The neurogenic progenitor cluster identified at P0 (Figure S1) was not detected at P7, and correlates with the expected downregulation of neurogenic genes (Ptf1a, Dcx, and Ascl1) at this age. Further clusters did not present a clear identity—based on known marker genes—and were therefore not considered for the analyses [file JNR-99-2228-s004.tif]

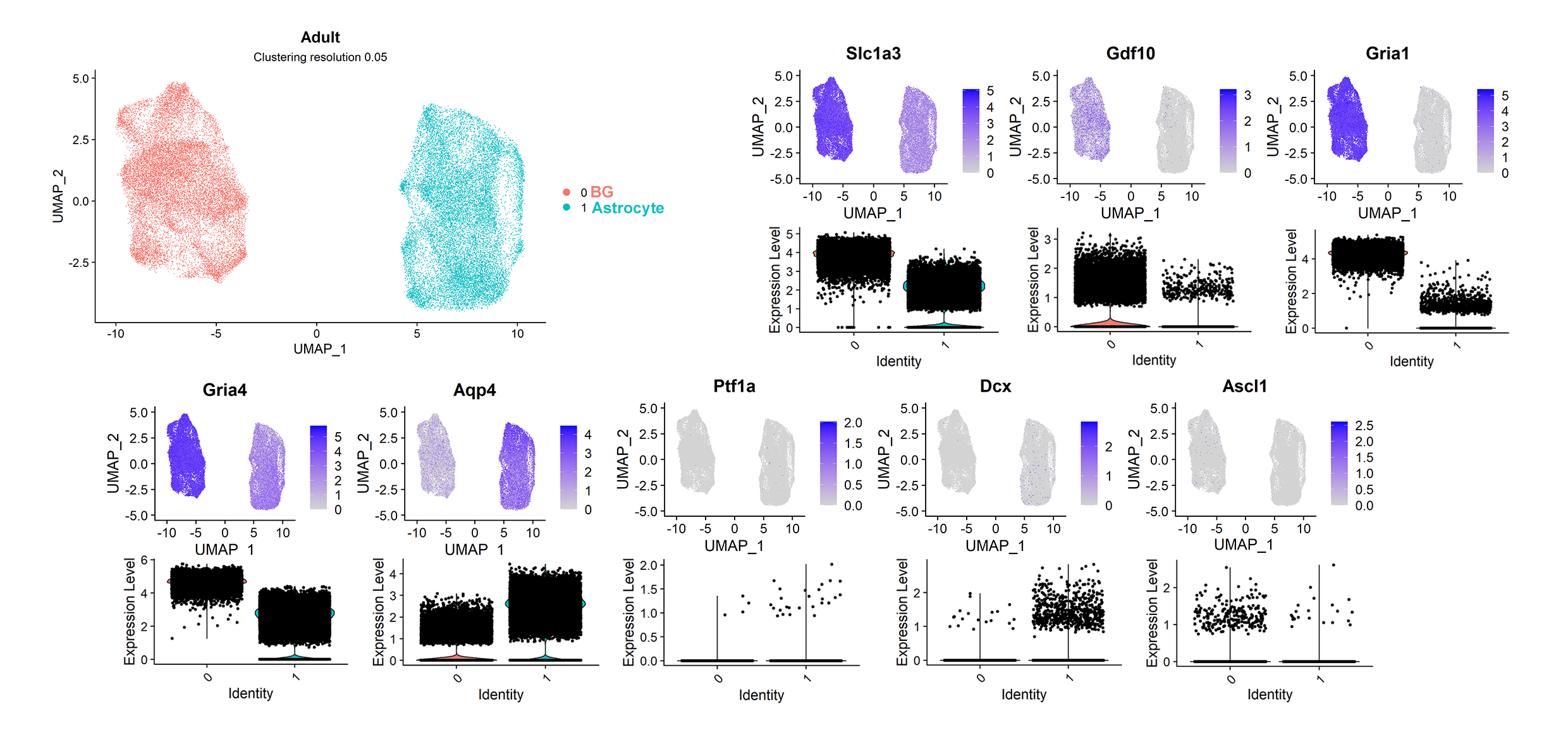

Supplement: Supplementary file 3 — FIGURE S3 Identification of distinct astrocyte subpopulations in snRNA‐seq data sets of adult cerebella. Seurat clustering of the astrocyte‐like cells in data set of a P60 (“Adult”) cerebella resulted in clearly segregated subpopulations that could be classified according to the expression of known marker genes (see Methods). The expression of BG‐enriched genes such as Gdf10, Gria1, and Gria4 allowed to identify the BG/BG progenitor subpopulations (cluster n. 0). The expression of Aqp4 was used to identify the parenchymal astrocytes/astrocyte progenitor population (cluster n. 1). The neurogenic progenitor cluster identified at P0 (Figure S1) was not detected in the adult cerebellum [file JNR-99-2228-s002.tif]

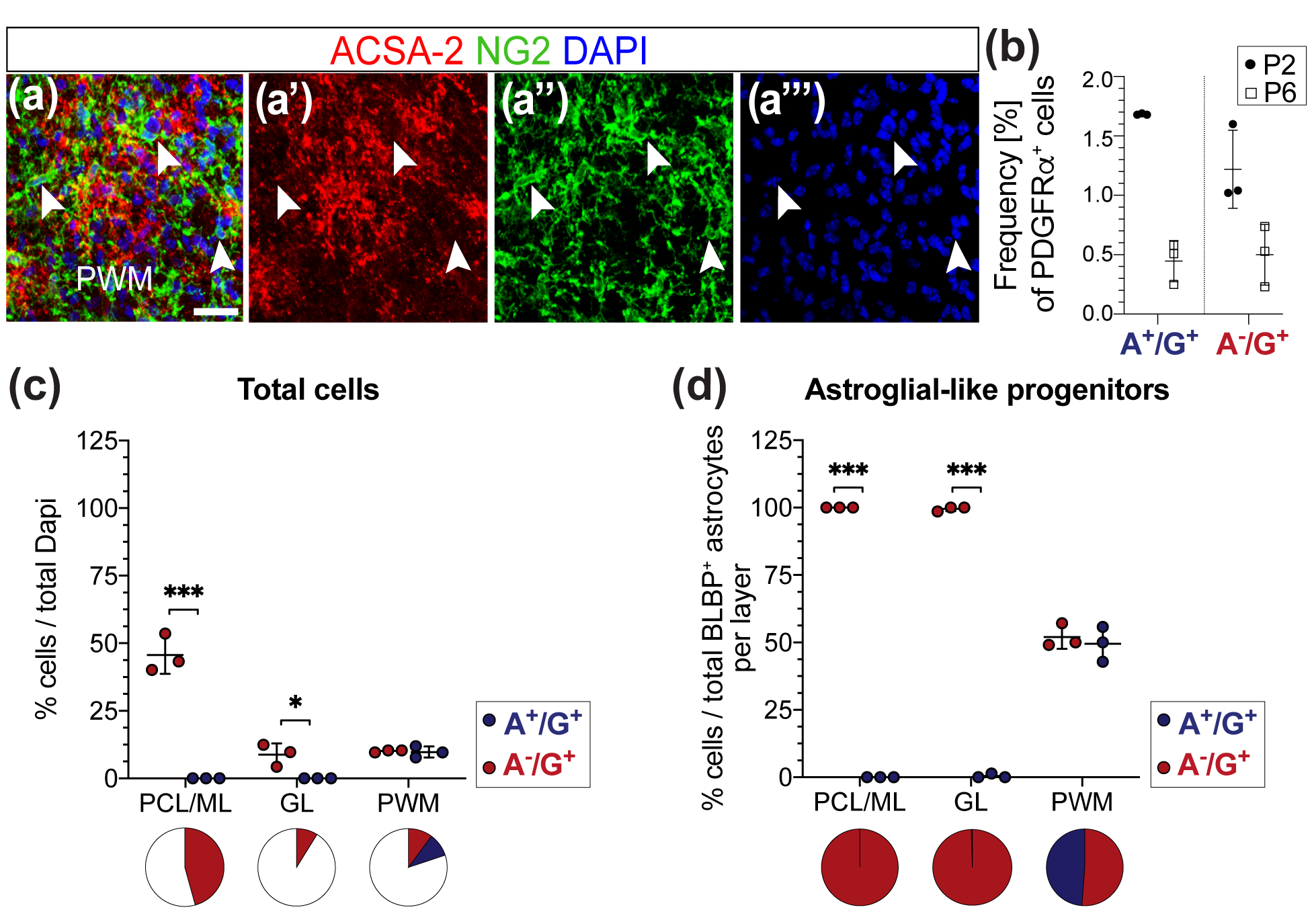

Supplement: Supplementary file 4 — FIGURE S4 ACSA‐2 is not expressed by neonatal cerebellar oligodendrocytes but is expressed by a subpopulation of astroglial‐like progenitors. (a) Confocal stacks of P3 cerebellum confirmed ACSA‐2 expression to be confined to cerebellar astrocytes in the PWM ACSA‐2 is not co‐expressed with the oligodendrocyte marker NG2 (a–a′″; filled arrowheads point to NG2+/ACSA‐2− cells). (b) Dissociated cerebellar samples were analyzed by flow cytometry for the co‐expression of ACSA‐2 and the oligodendrocyte precursor cell marker platelet‐derived growth factor receptor alpha (PDGFRα). The overlay was less than 2% for both populations at P1 ((A+/G+): 1.68%; (A−/G+): 1.22%) and at P3 ((A+/G+): 0.4517%; (A−/G+): 0.52%)). Frequencies of A−/G+ and A+/G+ cells described in Figure 2a–c were quantified over total cells (c) or total BLBP+ astroglial‐like progenitors (d) in the different layers of a P3 cerebella (i.e., PWM, GL, PCL/ML). Frequencies are presented also as pie charts for each cerebellar layer. At this stage A+/G+ cells are exclusively present in the PWM where they represent about half of the astroglial‐like progenitors. Scale bar: 100 μm. Nuclear stain: DAPI. GL, granular cell layer; ML, molecular layer; PCL, Purkinje cell layer; PWM, prospective white matter [file JNR-99-2228-s006.tif]

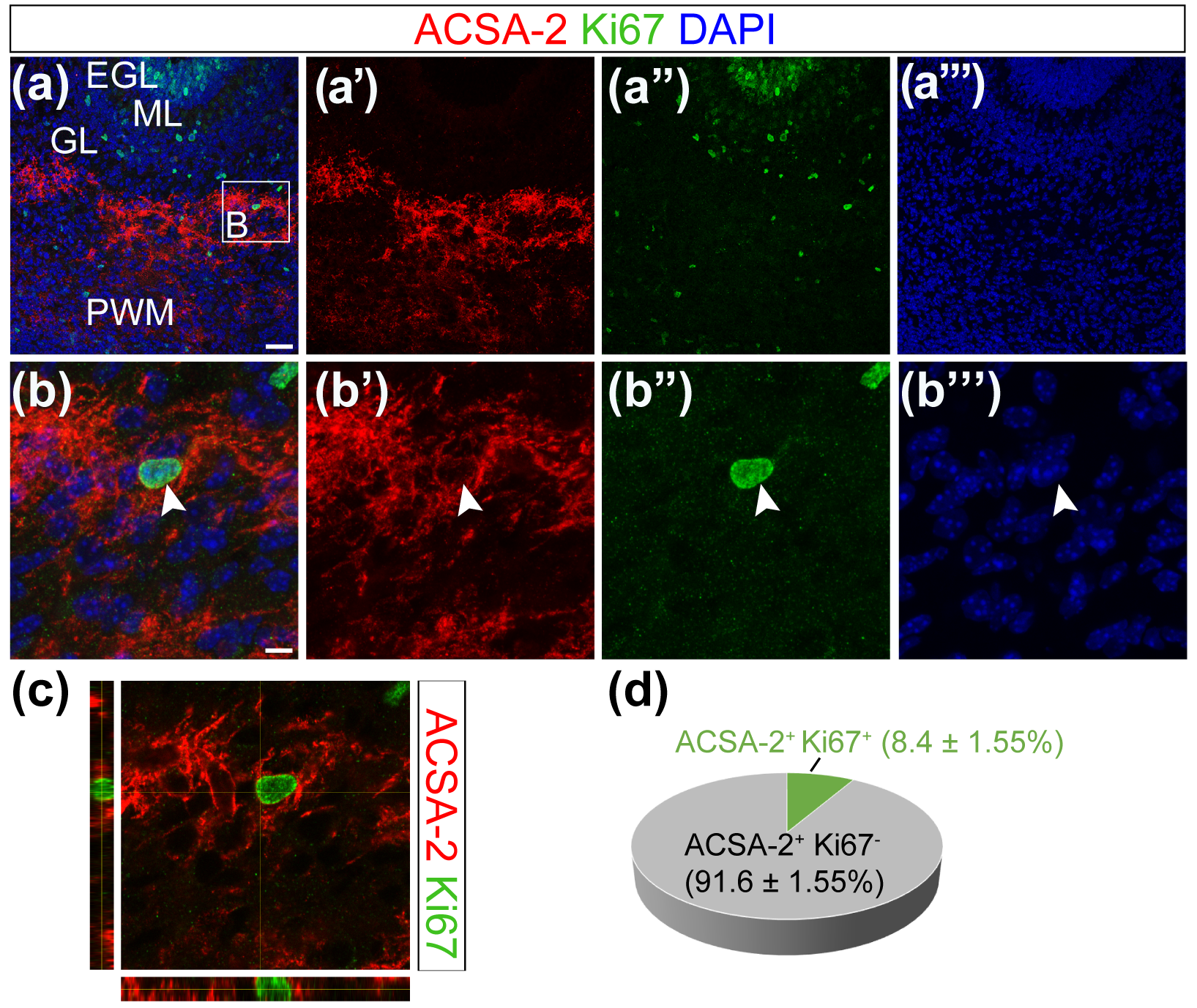

Supplement: Supplementary file 5 — FIGURE S5 A minor fraction of ACSA‐2+ precursors in the PWM proliferates. (a,b) Among the proliferating cells of the PWM a minor proportion of ACSA‐2+ cells ((d) 8.4% ± 1.6% (n = 3)) showed Ki67 positivity, thus proliferates (a and close‐up in b). Orthogonal projection shows the surface marker ACSA‐2 aligned around the Ki67+ nuclei (c). Scale bars: 30 μm (a,b). Nuclear stain: DAPI. EGL, external granular layer; GL, granular cell layer; ML, molecular layer; PWM, prospective white matter [file JNR-99-2228-s008.tif]

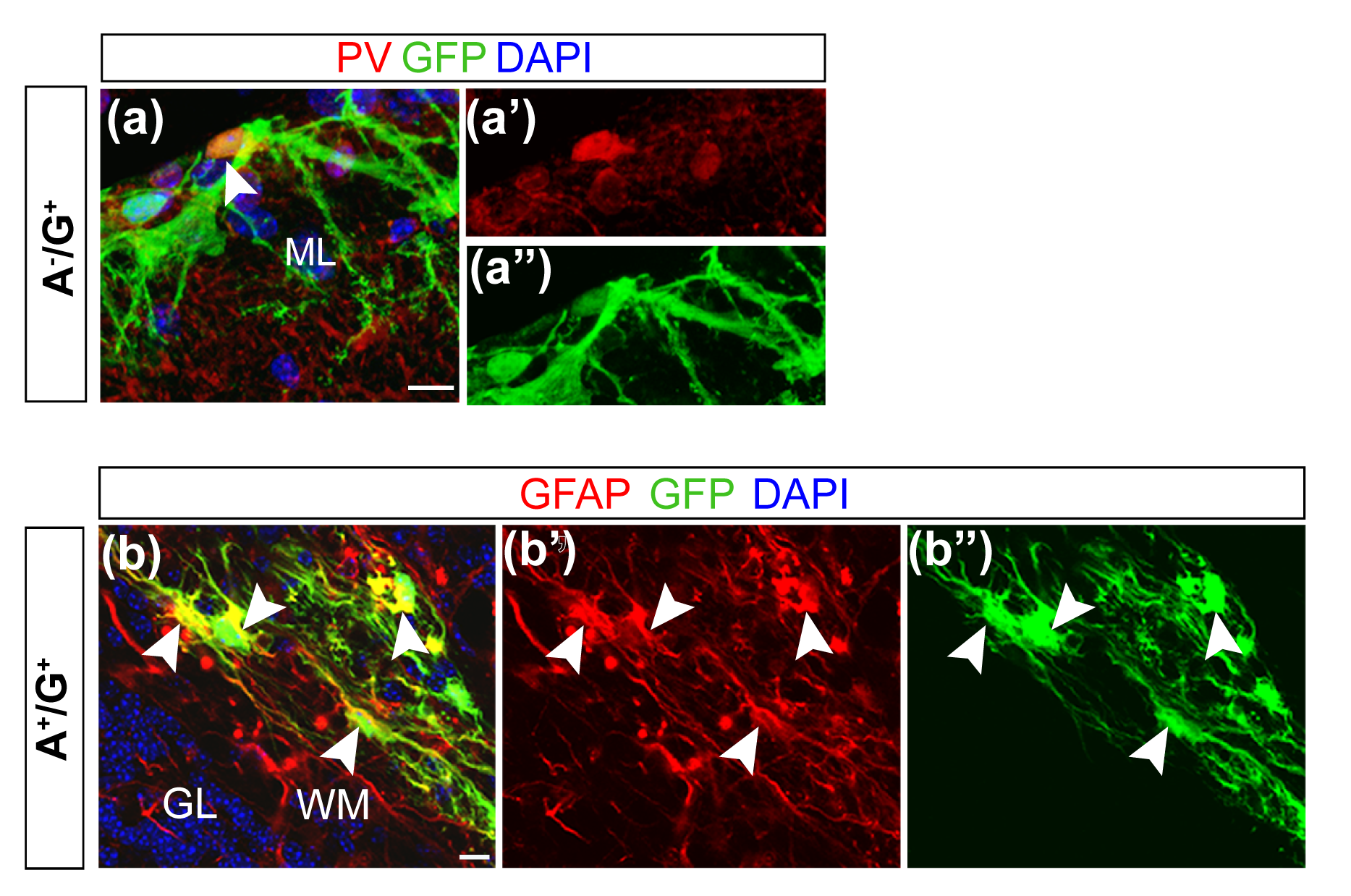

Supplement: Supplementary file 6 — FIGURE S6 A−/G+ precursors present a multipotent differentiation potential when transplanted into a non‐neurogenic environment. (a,b) A+/G+ and A−/G+ cells were isolated from β‐actin‐GFP+ cerebella and injected into the cerebellum of adult β‐actin‐GFP− mice (P60). (a) As seen by the co‐labeling of GFP (a,a″) and PV (a,a′) A−/G+ cells generate PV+ interneurons when transplanted into the adult cerebellum. (b) By contrast, A+/G+ cells differentiate exclusively into GFAP+ astrocytes as identified by the co‐staining of GFP (b,b″) with GFAP (b,b′). Scale bars: 10 μm (a,b) Sample group size: (a,b (n = 3)). GL, granular cell layer; ML, molecular layer; PV, Parvalbumin; WM, white matter [file JNR-99-2228-s005.tif]
